# Sterile Diet Causes Gut Microbiome Collapse of Cancer Patients Post Hematopoietic Cell Transplantation, But Normal Diet Recovers Them

**DOI:** 10.1002/advs.202403991

**Published:** 2024-07-08

**Authors:** Wenqing Hong, Yun Wu, Zimin Sun, Shu Yang, Qing Cheng, Huilan Liu, Xiaoxing Lin, Renjie Ni, Yuping Yao, Shuijing Wang, Zihao Zheng, Anyi Sun, Chuanwu Xi, Liyan Song

**Affiliations:** ^1^ School of Resources and Environmental Engineering Anhui University Hefei 230601 China; ^2^ Department of Hematology The First Affiliated Hospital of University of Science and Technology of China Hefei 230001 China; ^3^ Blood and Cell Therapy Institute Division of Life Sciences and Medicine Anhui Provincial Key Laboratory of Blood Research and Applications University of Science and Technology of China Hefei 230027 China; ^4^ Institute of Public Health Sciences Division of Life Science and Medicine University of Science and Technology of China Hefei 230026 China; ^5^ Department of Environmental Health Sciences University of Michigan Ann Arbor MI 48109 USA; ^6^ Chongqing Institute of Green and Intelligent Technology Chinese Academy of Science Chongqing 400714 China

**Keywords:** assembly mechanism, gut microbiome, leukemia patients, short‐chain fatty acids, sterile diet, umbilical cord blood transplantation

## Abstract

Though sterile diet, post‐transplantation surgery is a clinical strategy for patient care to prevent the infiltration of gut pathogens, less is known about its effects on the gut microbiome. Here, the gut microbiome dynamics of leukemia patients following a 120‐day “sterile‐normal” diet strategy posthematopoietic cell transplantation are examined. In contrast to the traditional idea, a sterile diet leads to the lowest gut microbiota diversity (*p* < 0.05) and short‐chain fatty acids, promoted the proliferation of potential pathogens such as *Streptococcus* (up by 16.93%) and *Lactobacillus* (up by 40.30%), and 43.32% reduction in nodes and an 85.33% reduction in edges within the microbial interaction's network. Interestingly, a normal diet allows the gut microbiome recovery and significantly promotes the abundance of beneficial bacteria. These results indicate that a sterile diet leads to a collapse of the patient's gut microbiome and promoted the proliferation of potential pathogens. This assay is a starting point for a more sophisticated assessment of the effects of a sterile diet. The work also suggests a basic principle for the re‐establishment of microbial equilibrium that supplementation of microbial taxa may be the key to the restoration of the degraded ecosystem.

## Graphical abstract

A sterile diet posthematopoietic cell transplantation damages the patient's gut microbiome, but a normal diet recovers them. This assay calls for a more sophisticated assessment of the effects of a sterile diet.

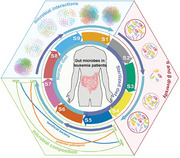

## 1 Introduction

Leukemia stands as the predominant cause of cancer among individuals under the age of 20 globally, comprising 40% of all cancer cases.^[^ ^1^, ^2^ ^]^ From 2007 to 2017, there was a 19% increase in the global incidence of leukemia, accompanied by a 13% increase in deaths linked to leukemia.^[^ ^1^, ^3^ ^]^ Allogeneic hematopoietic cell transplantation (allo‐HCT), specifically umbilical cord blood transplantation (UCBT), has emerged as one of the most widely used therapeutic methods in leukemia treatment.^[^ ^4^ ^]^ Post‐transplantation, patients are required to reside in a sterile environment and adhere to a sterile diet for a certain period,^[^ ^5^ ^]^ to prevent the infiltration of gut pathogens.^[^ ^6^ ^]^ Diet serves as one of the most effective regulators in reshaping the structure and function of the gut microbiota, which in turn can result in alterations in short‐chain fatty acids (SCFAs).^[^ ^7^ ^]^ Previous studies have established that dietary‐induced alterations in the gut microbiota as an important contributor to physiological processes and disease progression in the human body.^[^ ^8^ ^]^ The Ketogenic diet has the potential to extend lifespan and reduce disease incidence,^[^ ^9^ ^]^ the Paleolithic diet has been used for the treatment of inflammatory bowel disease,^[^ ^10^ ^]^ and the Mediterranean diet can lower overall mortality and the risk of various chronic diseases.^[^ ^11^ ^]^ Therefore, the sterile diet is supposed to impact the gut microbiome of post‐UCBT patients. However, this impact on the gut microbial dynamics of leukemia patients remains unknown.

“Human‐microbiota” and “microbe‐microbe” interactions follow intricate pathways to maintain a dynamic equilibrium.^[^ ^12^, ^13^ ^]^ Industrialized lifestyles are rapidly altering the associated microbiota and reshaping their equilibrium,^[^ ^14^ ^]^ contributing to the disappearance of crucial microbial taxa and diminishing the role of microorganisms in shaping human physiology.^[^ ^14^, ^15^ ^]^ This theoretical framework of microbial depletion helps to understand the increased incidence of various diseases. A significant viewpoint is that any disturbance to the microbiota may not only have transient effects but also lead to the extinction of certain microbial taxa, exerting cumulative effects on host physiology.^[^ ^16^ ^]^ Consequently, the theory of “rewilding” ancestral microbiota has emerged,^[^ ^17^ ^]^ yet there is a heated debate regarding whether rewilding is beneficial for humans or the environment, as there is a lack of theoretical or empirical consensus in these areas. One practical question within this debate is how the dynamics of microbial communities and their interactions change during the restoration process. The dynamics of the gut microbiome during the diet of leukemia patients post‐UCBT provide a reference for this fundamental question.

A 120‐day longitudinal study lasting was conducted at the First Affiliated Hospital of the University of Science and Technology of China, The study focused on a “sterile‐normal” diet strategy that comprised nine leukemia patients. The strategy consisted of a sterile diet for 30 days, followed by a normal diet for 90 days. The gut microbiota composition and dynamics were investigated and compared to those of the patient's healthy family members, who were used as a reference group. Results showed that the sterile diet causes the collapse of the gut microbiome and increased the abundance of potential pathogens. Interestingly, the normal diet helped recover the collapsed gut microbiota collapse and inhibited the proliferation of potential pathogens. These findings invite further evaluation of the effect of sterile diet's impact on patient health care. The recovery process after the gut microbiota collapse provides valuable insights for restoring microbial ecological balance for human patients and the deteriorated environment.

## 2 Result

### 2.1 Reshaping of the Gut Microbiota and SCFAs

**Figure 1:**
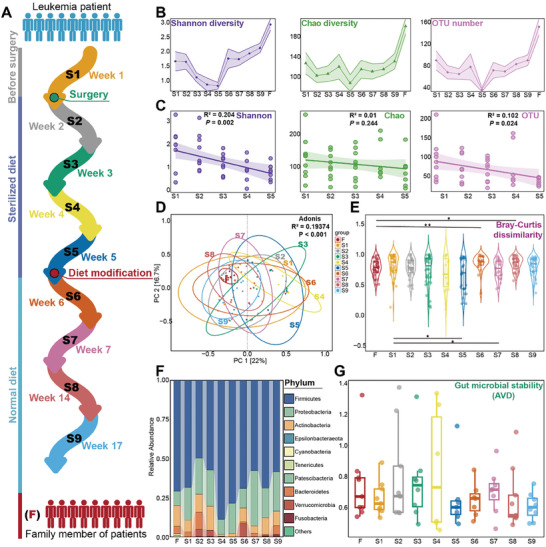
Experimental design and composition and diversity of bacterial community. A) Experimental design showing dietary interventions in nine leukemia patients from S1–S9, F represents the family members of each patient, who had no history of leukemia and were sampled concurrently at S9. B) Alpha diversity of the bacterial community. Shaded areas indicate the standard error of the mean. Statistical analysis was performed using the Wilcoxon test. C) Trend in α‐diversity of the bacterial community during the sterile diet. The shaded area around the smooth line represents the 95% confidence interval. Statistical analysis was conducted using a linear regression model with a two‐sided test and adjusted R‐squared was used. D) Principal Coordinates Analysis (PCoA) analyses of the bacterial community, analyzed using the Adonis test. E) Bray‐Curtis dissimilarity index, illustrating changes in β‐diversity of the bacterial community. Differences between S1 and other groups were examined using the Wilcoxon test. The figure only shows the results for F and S1 versus the other groups, with detailed test results available in Table S1 (Supporting Information). F) Relative abundance of the bacterial community at the phylum level. Only the top ten phyla were retained, with the remaining phyla combined as “others.” G) Stability index average variation degree (AVD) of gut microorganisms. A lower AVD indicates a more stable community(*n* = 83).

Leukemia patients typically undergo the “sterile‐normal” diet transition following UCBT.^[^ ^5^, ^6^ ^]^ During this dietary intervention, it is anticipated that the gut microbial community of the patients will undergo substantial changes. To validate this hypothesis, a comprehensive analysis of the bacterial community diversity was conducted. The Shannon index, Chao index, and OTU number displayed similar trends over the time series: a decrease during S1–S5 (sterile diet), followed by an increase during S5–S9 (normal diet) (**Figure** **1**B). Meanwhile, patients exhibited lower gut microbial diversity compared to family members (Figure 1B). During the sterile diet, both the Shannon index (*p *= 0.002) and OTU number (*p *= 0.024) showed a significant decrease (Figure 1C). Interestingly, we observed that the three indices were more dispersed among different patients at S1 due to antibiotics application before surgery, but by S9, they showed greater convergence. Conversely, individual differences were smaller in the family members group than in patients (Figure 1E). Furthermore, similar conclusions were drawn from the analysis of β‐diversity (Figure 1D).

Specifically, during the S1–S5 period, individual differences in the gut bacterial community increased significantly (Figure 1D) (*p* < 0.001, Adonis test). In contrast, during the S6–S9 period, the 95% confidence ellipses displayed a shrinking trend (Figure 1D) (*p* < 0.001, Adonis test). Additionally, the Bray‐Curtis dissimilarity index was calculated for each sample to characterize the β‐diversity.^[^ ^18^ ^]^ During the S2–S4 period, the dissimilarity index remained dispersed. However, at S5, the dissimilarity index converged (Figure 1E), suggesting that S5 may represent a turning point. To further investigate this phenomenon, the average variation degree (AVD) index was used to measure the stability of bacterial communities.^[^ ^19^ ^]^ Lower AVD values indicate greater stability of the microbial community. The gut microbiome became increasingly unstable during the S1–S4 period (Figure 1G). However, at S5, it appeared to reach a new steady state, with greater similarity among individuals. During the S6–S9 period, the gut microbiota underwent another transition from instability to stability (Figure 1G). Notably, the individual differences in the gut microbiota were much smaller during the normal diet than during the sterile diet, as confirmed by the dissimilarity index (Figure 1E).

Besides the substantial changes in gut microbiota, the sterile diet caused fluctuations in SCFA levels. The trend in acetic acid levels mirrored the changes in diversity (Figure S2, Supporting Information; Figure 1B). Notably, acetic acid, butyric acid, isovaleric acid, isobutyric acid, propionic acid, and valeric acid levels were all at their lowest levels at S5, suggesting an unhealthy state of gut microbiota under the sterile diet.

### 2.2 Composition of the Gut Microbiota

**Figure 2:**
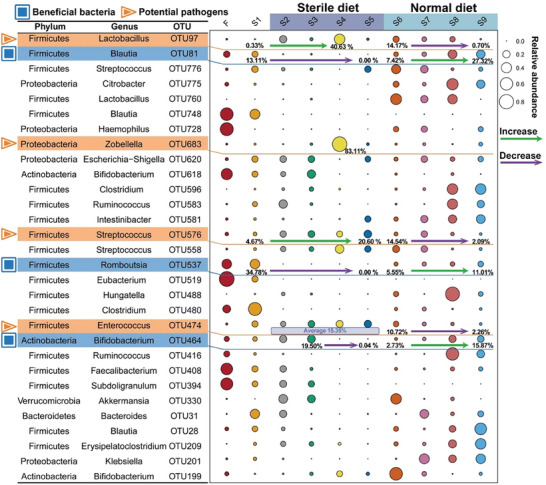
Trends in relative abundance in the top 30 OTUs during dietary intervention, *n* = 83.

The phylum Firmicutes was the most abundant group, with an average proportion of 64.6 ± 29.9% during S1–S9, and 70.8 ± 19.2% in the family members (Figure 1F). To gain a more detailed understanding, we visualized the top 30 most abundant OTUs (**Figure** **2**). Interestingly, the relative abundances of OTU 81 (genus *Blautia*), OTU 537 (genus *Romboutsia*), and OTU 464 (genus *Bifidobacterium*) were noticeably higher during the normal diet. During the sterile diet, their relative abundances were generally low and showed a decreasing trend. These bacteria are known to be beneficial to the human body^[^ ^20^, ^21^, ^22^ ^]^ but appear to repopulate only when a normal diet is resumed. Conversely, OTU 683 (genus *Zobellella*), OTU 97 (genus *Lactobacillus*), OTU 576 (genus *Streptococcus*), and OTU 474 (genus *Enterococcus*) increased during the sterile diet and gradually decreased during the normal diet (Figure 2). Though *Lactobacillus* has many reported benefits for the human body, studies have shown that it can increase the risk of infection in immunocompromised individuals.^[^ ^23^ ^]^ Genus *Streptococcus* and *Enterococcus* can cause various infections or be associated with the development of various metabolic disorders.^[^ ^24^, ^25^ ^]^ Furthermore, OTU 474 (*p* < 0.05) showed a significant positive correlation with the sterile diet, while OTU 81 (*p *< 0.05) and OTU 464 (*p* < 0.05) showed a significant negative correlation with the sterile diet (Figure S3, Supporting Information, Spearman test).

The alteration of gut microbiota composition affects the production of SCFAs derived from fermented dietary fibers. Therefore, Spearman correlation analysis was used to examine the relationship between the top 30 most abundant OTUs and SCFAs. Specifically, OTU 464 (genus *Bifidobacterium*, *p* < 0.05), OTU 394 (genus *Subdoligranulum*, *p* < 0.05), and OTU 519 (genus *Eubacterium hallii*, *p* < 0.05) exhibited strong relationships with butyric acid (Figure S4A, Supporting Information, Spearman test). Additionally, significant correlations were found between bacterial community composition and acetic acid, propionic acid, valeric acid, and butyric acid (Figure S5, Supporting Information, *p* < 0.05, Spearman test). Furthermore, a significant correlation was observed between butyric acid and bacterial community Shannon index (*p* < 0.05) (Figure S5, Supporting Information, Spearman test).

### 2.3 Gut Microbial Interactions

**Figure 3:**
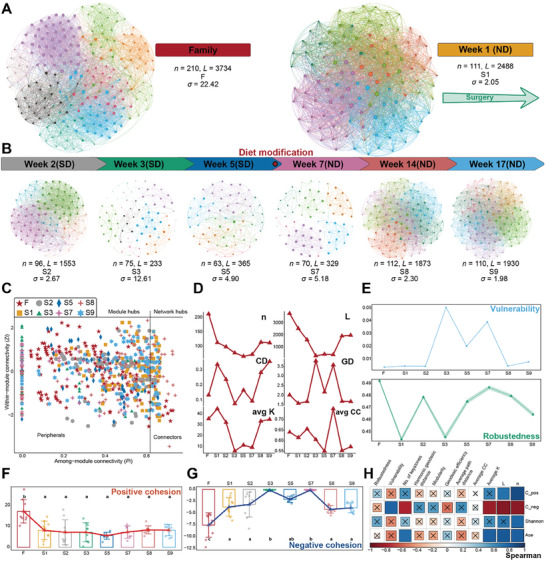
Gut microbial community interactions. The family members and patients S1 are A). After surgery, the patients go through the stages of the “sterile‐normal” diet B), colors indicate module membership. ND denotes normal diet; SD denotes sterile diet; σ denotes the characteristics of a small world, with σ > 1, i.e., the existence of a “small world”; n and L denote the number of nodes and edges of each network. C) The distribution of key species. D) Network topology. Centralization of degree (CD), average path distance (GD), average degree (avgK), and average clustering coefficient (avgCC). E) The change in vulnerability and robustness, shaded areas indicate standard error of the mean. F,G) Positive and negative cohesion, respectively. Different lowercase letters indicate statistics significant differences. H) The correlation test between the network topology and the correlation test between community characteristics, “×” indicates nonstatistics significance (*p* > 0.05), Spearman test. C_pos denotes positive cohesion, and C_neg denotes negative cohesion.

We constructed 8 Microbial Ecological Networks (MENs)based on the logarithmic transformation of OTU abundances (**Figure** **3**A,B) using the Random Matrix Theory (RMT) method.^[^ ^26^, ^27^ ^]^ The RMT method can keep the maximal OTUs interaction in the MENs. Due to the requirement for an adequate number of samples, we excluded S4 and S6. This exclusion does not affect our overall assessment of the temporal trends in MENs. However, caution should still be exercised when interpreting the mechanisms underlying these networks.

All networks exhibit “small‐world” characteristics (σ > 1, Figure 3A,B).^[^ ^28^ ^]^ No isolated nodes were observed in any of the MENs. The total nodes (n) were reduced by 43.2%, and the total edges (L) were reduced by 85.3% during the sterile diet. However, as indicated by the diversity results, S5 represented a steady state of the sterile diet treatment with an increased number of edges (Figure 3B). During the normal diet (S7–S9), the interaction network of gut microbiota likely collapsed at S7 but gradually recovered thereafter. This result strongly reflects the drastic changes in the gut microbiota, with the total nodes increasing by 57.1%, and the total edges increasing by 486.6% during normal diet. Positive correlations among nodes primarily represent cooperative behaviors, while negative correlations indicate competition for limited resources and ecological niche overlap.^[^ ^29^ ^]^ The proportion of positive correlations rapidly increased from S1 (59.7%) to S3 (99.6%), then decreased at S5 (88.5%), and continued to decline from S7 (99.7%) to S9 (62.8%) (Figure S6, Supporting Information).

These changes further influenced the organizational principles of the network. Both average degree (avgK) and centralization of degree (CD) exhibited trends similar to diversity (Figures 1B and 3D), with avgK and CD reflecting the average number of connections per node and the distribution of degrees in the network, respectively. Positive cohesion represents the degree of cooperative behavior within the community, and negative cohesion indicates competition between communities, reflecting the degree of mutual exclusion between nodes.^[^ ^30^ ^]^ Both positive and negative cohesion showed significant differences between F and S1–S9 (*p* < 0.05, Tukey test) (Figure 3F,G), indicating differences in gut microbial interactions between normal and leukemic individuals. The negative cohesion of S3 and S7 was close to 0, indicating lower competition. Additionally, negative cohesion was significantly negatively correlated with the number of keystones, avgK, L, and n in the network (Figure 3H, Spearman test).

## 3 Discussion

The microbiota, acting as a bridge between dietary patterns and human health,^[^ ^31^ ^]^ plays a pivotal role as a driving force in maintaining the host's health.^[^ ^16^ ^]^ Traditionally, a sterile diet following transplant surgery has been a clinical strategy for patient health. Understanding the link between sterile diet and host gut microbiome holds significant implications for patient health management. In this study, we examined the gut microbiota dynamics in leukemia patients and presented compelling evidence from multiple perspectives that the sterile diet consistently disrupts the equilibrium of the patient's gut microbial, leading to the proliferation of potential pathogens. Additionally, the concentrations of all determined SCFAs reached their lowest levels at the end of the sterile diet. However, a normal diet allows the recovery of gut microbial ecological balance (microbiota diversity, composition, and interactions) and inhibits the proliferation of potential pathogens.

A sterile diet led to a collapse of the patient's gut microbiota diversity and balance. There was a significant correlation between the sterile diet and the diminishment of gut microbiota diversity (Figure 1C and *p* < 0.05). Although the gut microbiota appeared to reach a novel state of equilibrium at S5 (Figure 1G), this state markedly differed from that of healthy individuals (Figure 1B,E,G), and this homeostasis was further disrupted with subsequent normal diet treatment (Figure 1B,G). Recent investigations have underscored that dysbiosis of the gut microbiota can further compromise the immune system function of patients, with inflammatory diseases being the most prevalent manifestations resulting from gut microbiota imbalance.^[^ ^32^, ^33^ ^]^ For patients, the diminishment of gut microbiota diversity may amplify the potential peril of pathogenic infections.

A sterile diet promotes the proliferation of potential pathogens. During the sterile diet, we observed a significant proliferation of specific pathogenic microbial communities, while the relative abundance of beneficial bacteria declined. For example, genera *Blautia* (*p* < 0.05), *Romboutsia*, and *Bifidobacterium* (*p* < 0.05), known to be beneficial for the human body,^[^ ^20^, ^21^, ^22^ ^]^ all exhibited a declining trend during sterile diet (Figure S3, Supporting Information, Figure 2). The sterile diet also promoted the relative abundances genera *Zobellella* (83.1% at S4), *Lactobacillus* (increased 40.3%), *Streptococcus* (increased 17.0%), and *Enterococcus*, (average 15.4%) (Figure 2; Figure S3, Supporting Information), and these OTUs are often sources of infections in immunocompromised individuals.^[^ ^23^, ^24^, ^25^ ^]^ These results suggest that a sterile diet increases the risk of infection, counteracting its intended purpose. Kamada et al.^[^ ^34^ ^]^ revealed that pathogens have evolved different strategies to overcome colonization resistance. One important strategy is to promote host inflammation to gain a growth advantage,^[^ ^35^ ^]^ thereby impeding the survival of commensal bacteria. This not only reduces competition from other bacteria but also provides more opportunities for pathogen colonization and proliferation.^[^ ^36^ ^]^

A sterile diet leads to a progressive simplification of interactions among gut microbes. Like diversity, the number of network nodes (reduced by 43.2%), edges (reduced by 85.3%), negative correlations (reduced by 40.3–11.5%), and certain network topological features (e.g., avgK and CD) all showed a declining trend during the sterile diet (Figure 3; Figure S6, Supporting Information), indicating that sterile diet led to a simplification of the network's organizational structure. These network characteristics demonstrated an increasing trend during normal diet, implying that a normal diet promotes interactions among intestinal microbiota. A diverse gut microbiota with well‐established interactions contributes to immune system enhancement, improving the body's ability to resist pathogens, and inhibiting the growth of potential pathogens.^[^ ^37^, ^38^ ^]^ Thus, the simplified microbial network structure caused by a sterile diet increased the risk of pathogen infections.

Additionally, a sterile diet results in disruption of the gut microbiota and may impair the normal production of SCFAs, leading to abnormal fluctuations in SCFA levels within the patient's body. Notably, these levels reach their lowest point on S5. The disruption of gut microbiota balance results in profound changes within the patient's body, including the intricate interplay among gut microbial communities. In this regard, butyric acid, as a significant intermediary in controlling microbial community, host metabolism, and energy homeostasis, can enhance intestinal barrier function to reduce inflammation and improve metabolic health.^[^ ^39^, ^40^ ^]^ The genera *Bifidobacterium*, *Subdoligranulum*, and *Eubacterium hallii* are known to have positive effects on butyric acid production.^[^ ^41^, ^42^, ^43^ ^]^ However, in the collapsed gut ecosystem, these microorganisms do not appear to promote butyric acid production (Figure S4, Supporting Information, *p* < 0.05).

Interestingly, a normal diet recovered the microbial diversity, composition, and interactions. As the stability of the gut microbiota increased (Figure 1G), the microbial diversity gradually recovered (Figure 1B), and the microbial interactions became more intensive (Figure A,B,D). Importantly, the relative abundance of potential pathogens decreased such as the genera *Lactobacillus* (decreased 13.5%), *Streptococcus* (decreased 12.5%), and *Enterococcus* (decreased 8.5%),^[^ ^23^, ^24^, ^25^ ^]^ while beneficial microbes such as the genera *Blautia* (increased 20.0%), *Romboutsia* (increased 5.5%), and *Bifidobacterium* (increase 13.1%)^[^ ^20^, ^21^, ^22^ ^]^ became more abundant (Figure 2; Figure S3, Supporting Information). These results suggest that a normal diet could remediate the microbial imbalance caused by a sterile diet.

In summary, the results from the perspectives of gut microbial diversity, composition, and interactions all indicated that a sterile diet disrupts the balance of the patient's gut microbiome, rendering them less capable of resisting external pathogens. In contrast, a normal diet significantly prevents the infiltration of external pathogens through the establishment of a balanced gut microbiota. The underlying mechanism may be that the unbalanced gut microbiome promotes the proliferation of potential gut pathogens^[^ ^35^, ^44^ ^]^ and a more diverse microbiome protects against pathogen colonization in the gut.^[^ ^44^ ^]^ The finding represents a starting point for a more sophisticated assessment of the effects of a sterile diet.

Specifically, the sterile diet represents only supplemental energy and nutrients, whereas the normal diet represents supplemental energy, nutrients, and microbial taxa in the restoration of a deteriorated microbial ecosystem. In this context, restoring the ecological balance of the microbiota is the primary objective. Here, We propose that during the re‐establishment of microbial equilibrium, through fecal microbiota transplantation, microbiome therapeutics, and rewilding microbiomes,^[^ ^17^, ^45^, ^46^ ^]^ supplementation of microbial taxa may be the key to the restoration of intestinal health and remediation of contaminated environments.

## 4 Experimental Section

### Ethics Statements

The fecal sample protocol was approved by the Institutional Animal Care and Use Committee of Chongqing Institute of Green and Intelligent Technology, Chinese Academy of Sciences (Approval ID: ZKCQY0066). The data and code were publicly stored in GitHub (https://github.com/hongwenqing/‐gut‐microbiome‐of‐cancer‐patients.git).

### Experimental Design and Sampling

This study was conducted at the Blood and Cell Therapy Institute of the First Affiliated Hospital of the University of Science and Technology of China (FAHUSTC), China. This Blood and Cell Therapy Institute is the largest cord blood transplantation surgery center in Asia. All patients consented to this experiment.

Fecal samples were collected from 9 leukemia patients at weekly intervals, starting from the week before cord blood transplantation surgery until the recovery process. The sampling timeline was as follows: Week 1 (S1), week 2 (S2), week 3 (S3), week 4 (S4), week 5 (S5), week 6 (S6), week 7 (S7), week 14 (S8), and week 17 (S9). Additionally, fecal samples were collected from nine family members of the patients (F, one family member per patient). The patients underwent cord blood transplantation surgery between the first and second weeks, with the first week being the preoperative period. Weeks 2–5 represented the 4 weeks after the patient's surgery ended, during which they lived in a sterile environment and adhered to a sterile diet. From the sixth week onward, the patients resumed a normal diet (Figure 1A; Figure S1, Supporting Information), sterile diet in this study is defined as food that is free from any microorganisms after treatment by autoclave sterilization method. Fecal samples were collected while wearing sterile gloves to prevent contamination. Approximately 20–100 g of feces were divided into three sterile sample cups and covered with lids. The samples were immediately placed in a −80 °C freezer until further use (two ice packs were placed inside a transport box, and the samples were stored in a −80 °C freezer within 5 min). Few samples could not fulfill sequencing requirements due to insufficient sample volume, and these samples could not be replenished due to their timeliness, see Table S2 (Supporting Information) for specific missing samples (Table S2, Supporting Information). Ultimately, a total of 83 samples were included in the sequenced analysis, and 58 samples were measured for SCFAs.

### Patient Source

Selected malignant hematological diseases who underwent single unrelated cord blood transplantation (UCBT) with the incomplete concordance of unrelated Human Leukocyte Antigen at the First Hospital Affiliated with the University of Science and Technology of China (Anhui Provincial Hospital) from 28 February 2018 to 27 March 2018 There were nine patients, three males, and six females, with a median age of 13 (3–34) years. The median age was 13 (3–34) years. Disease type: acute lymphoblastic leukemia (ALL) in six cases and acute myeloid leukemia (AML) in three cases. Pretransplant disease status: four cases in first complete remission (CR1), three cases in second complete remission (CR2), and two cases in null remission (NR). All of them were treated with a regimen of clear marrow preconditioning myeloablative conditioning (MAC) and without antithymocyte globulin (ATG) for the prevention of graft‐versus‐host‐disease (GVHD) (Flu30mg/m^2^×4+Bu 0.8 mg kg^−1^ q6h×4d+CY60mg/ kg×2d). Implantation was obtained in all patients after transplantation. The median time to neutrophil implantation was 18 (14–22) days and platelet implantation was 34 (19–41) days. A sterile diet was given from pre‐treatment till 30 days post‐transplantation and a normal diet was given 30 days post‐transplantation. Patients were voluntarily enrolled to retain stool specimens. Follow‐off time: 2 November 2023, six out of nine patients in this group survived with a median survival time of 68 (5–69) months, and three patients died (1 cerebral hemorrhage, 1 fungal infection of the lungs, and 1 recurrence of the original disease), as shown in Table S3 (Supporting Information).

### Short‐Chain Fatty Acids Determination

Fecal SCFAs were determined by gas chromatography‐mass spectrometry (GC‐MS). The samples were first derivatized.^[^ ^47^ ^]^ Briefly, 200 mg fresh weight feces were homogenized with 1 mL 10% isobutanol and centrifuged at 6000 x g for 20 s twice with a 30 s interval. Samples were then centrifuged at 10 000 x g for 5 min and 675 uL supernatant was transferred to a new tube with 20 ug 3‐methyl pentanoate added as internal standard. The 125 uL 20 mm NaOH solution and 400 uL chloroform were added to the tube. After brief vortexing, the tube was centrifuged at 10 000 x g for 2 min and 400 uL supernatant was transferred into a new tube. Then, 70 uL isobutanol, 100 uL pyridine, and 80 uL ultra‐pure water were added to the tube in sequence. At the same time, zeolite particles were added to the tube to avoid bumping. The 50 uL isobutyl chloroformate was carefully added to the tube and some gases were generated. The tube was left open for 1 min to allow the release of the generated gases. The tube was then closed and vortexed for 2 min, after which 150 uL hexane was added, followed by centrifugation at 10 000 x g for 2 min. Thereafter, the extracted supernatant was measured by GC‐MS using the Agilent 7890A (Agilent Technologies, CA, USA) coupled to the Agilent 5977A mass selective detector.

The sample was injected into GC‐MS in a split mode (1:50) with an injection volume of 1 uL. HP‐5MS (30 m × 0.25 mm × 0.25 um) was used as the GC column. The solvent delay was 5 min. The temperature of the transfer line and the GC‐MS ion source were both set to 250 °C. The initial oven temperature was 40 °C, maintained for 5 min, then programmed to increase to 150 °C at a rate of 5 °C min^−1^, and finally to 300 °C at 40 °C min^−1^. The retention times of acetic acid, propionic acid, butyric acid, isobutyric acid, valeric acid, isovaleric acid, and 3‐methyl pentanoic acid were 5.00, 8.46, 11.79, 10.24, 15.22, 13.46, 17.08 min, respectively. The limits of detection (LODs) and limits of quantification (LOQs) were in the range of 0.05–5.00 g L^−1^. The linear regression coefficients (R2) for all standard acid solutions were higher than 0.9955.

### Genomic DNA Extraction and PCR Amplification

Genomic DNA extraction was performed using the Power Soil DNA Isolation Kit (MoBio, USA) according to the manufacturer's instructions. The 1% agarose gel electrophoresis was used to check the extracted genomic DNA.^[^ ^48^ ^]^ The genomic DNA was stored and transported to Majorbio Bio‐pharm Technology Co., Ltd (Shanghai, China) at −20 °C for sequencing. The amplification of V3‐V4 16S rRNA gene regions was conducted using primers with 338F/806R. PCR was performed using TransGen AP221‐02 (TransStart Fastpfu DNA Polymerase) on an ABI GeneAmp (9700 model). The amplification procedure was as follows: predenaturation at 94 °C for 3 min; followed by 25 cycles (denaturation at 95 °C for 30 s, annealing at 57 °C for 30 s, and extension at 72 °C for 45 s), and final 10 min extension at 72 °C. The amplicons were purified with AxyPrep DNA Gel Extraction Kit (Axygen, USA) and used to generate the libraries using TruSeq DNA Sample Prep Kit (Illumina, USA). The libraries were sequenced on the Illumina MiSeq platform with a 250‐bp paired‐end strategy. The raw data were submitted to the National Center for Biotechnology Information (NCBI) under project number PRJNA1030792.

### Sequence Data Optimization and OTU Clustering

The paired‐end reads were assigned to each sample based on the unique barcode, then these reads were trimmed and assembled by using FLASH (v1.2.11). The sequences were then quality‐controlled and filtered using QIIME (v1.9.1). After the sample assignment, Operational Taxonomic Unit (OTU) clustering analysis and taxonomic classification were performed. USEARCH (http://drive5.com/usearch/) was used to extract non‐redundant sequences from the optimized sequences, and singletons were removed. The nonredundant sequences were clustered into OTUs at a 97% similarity level, and chimeras were removed during the clustering process to obtain representative sequences for the OTUs. To obtain taxonomic classification information for each OTU, the RDP classifier (Release 11.5 http://rdp.cme.msu.edu/) Bayesian algorithm was used to perform taxonomic analysis on the representative sequences of the OTUs at the 97% similarity level.

### α and β‐Diversity

Using the R 3.6.3 and R Studio 1.1.463 platforms, the α‐diversity indices of the gut bacterial community, including the Chao 1 and Shannon diversity indices, were calculated using the “vegan” package. The non‐parametric Wilcoxon test was used to compare the differences in α‐diversity between S1 and other groups.

Additionally, using the “vegan” package, principal coordinates analysis (PCoA) based on the Bray–Curtis distance and Adonis test was performed to compare the β‐diversity of the gut bacterial community at different sampling points. The differences in PC1 and PC2 between S1, S9, and F were tested using the Tukey test.

### Community Stability and Molecular Ecological Networks

The average variation degree (AVD) was calculated in R 3.6.3 and RStudio 1.1.463 platforms to measure the stability of the gut microbial community (Equation 1), in which k is the number of samples in one sample group, n is the number of OTUs in each sample group, *x_i_* is the rarefied abundance of the OTU in one sample, ${\bar{x}}_{i}\;$ is the average rarefied abundance of the OTU in one sample group, and δ_*i*_ is the standard deviation of the rarefied abundances of the OTU in one sample group. A lower AVD value indicates a more stable community.^[^ ^19^ ^]^

(1) $$\mathrm{AVD}\;=\;\frac{\sum_{i\;=\;1}^{n}\frac{\left|x_{i}-{\bar{x}}_{i}\right|}{\delta_{i}}}{k\;\times n}$$

All MENs were constructed based on the RMT method using log‐transformed OTU abundances.^[^ ^26^, ^27^ ^]^ RMT predicts two universal extreme distributions of the nearest neighbor spacing distribution (NNSD) of the eigenvalues: Gaussian orthogonal ensemble (GOE) statistics, which corresponds to the stochastic nature of the complex system, and the Poisson distribution, which corresponds to the system‐specific non‐stochastic nature of the complex system. RMT's approach assumes the existence of a transition point from the GOE to the Poisson distribution, which can be used as a threshold for defining the adjacency matrix. This transition point mathematically determines the number of OTUs used to build the network. Furthermore, this method effectively removes noise from nonrandom features.^[^ ^27^, ^49^ ^]^ The integrated Network Analysis Pipeline (iNAP) tool, based on the RMT method, was used for network construction^[^ ^30^, ^50^, ^51^ ^]^ and the networks were visualized using Gephi (0.9.2).

The network topological parameters, such as n, L, CD, GD, avg K, avg CC, vulnerability, and robustness, were computed using the iNAP platform (http://mem.rcees.ac.cn:8081/). The specific meanings of these indices can be found in the figure caption. For each network node, its within‐module connectivity (Z_i_) and among‐module connectivity (P_i_) were calculated to classify its role in the network. Nodes with Z_i_ ≥ 2.5 and *P*_i_ < 0.62 are referred to as module hubs, while nodes with Z_i_ < 2.5 and *P*_i_ ≥ 0.62 are connectors. Nodes with Z_i_ ≥ 2.5 and *P*_i_ ≥ 0.62 are considered network hubs, and these nodes are known as keystone nodes.^[^ ^52^, ^53^, ^54^ ^]^ The values of Z_i_ and *P*_i_ were also computed using the iNAP platform. The positive or negative cohesion was calculated using the method proposed by Yuan^[^ ^30^ ^]^ in R language, and a Spearman correlation heatmap was generated using the “corrupt” package to depict the relationships between network topological parameters and community diversity.

### The Correlation Between the Characteristics of the Gut Microbiota and SCFAs

The putative bacterial functional composition was predicted using PICRUSt2, based on the Kyoto Encyclopedia of Genes and Genomes (KEGG) database (http://www.genome.jp/kegg/), which provides information on the gut microbiota's functional composition to some extent. The correlation between OTUs, KO, fatty acids, and community α‐diversity indices was evaluated using the Mantel test in R, with the “LinkET” package.

## Conflict of Interest

The authors declare no conflict of interest.

## Author Contributions

W.H. and Y.W. contributed equally to this work. L.S., W.H., Y.W., Z.S., and C. X. performed conceptualization. W.H., L.S., S.Y., Q.C., H.L., R.N., X.L., S.W., Y.Y., Z.Z., and A.S. performed methodology. Y.W., S.Y., Q.C., R.N., X.L., S.W., Y.Y., Z.Z., and A.S. performed investigation. W.H., Y.W., and S.Y. performed visualization. L.S., and S.Y. performed Funding acquisition and project administration. Y.W., L.S., Z.S., S.Y., and H.L. performed supervision. W.H., L.S., and Y.W. wrote original draft. L.S., Z.S., S.Y., and C.X. wrote reviewed and edited.

## Supporting information

Supporting Information

## Data Availability

The data that support the findings of this study are available on request from the corresponding author. The data are not publicly available due to privacy or ethical restrictions.
