# Supplementary material for: Sterile Diet Causes Gut Microbiome Collapse of Cancer Patients Post Hematopoietic Cell Transplantation, But Normal Diet Recovers Them
Source: Adv Sci (Weinh). 2024 Jul 8;11(34):2403991. doi: 10.1002/advs.202403991 (PMC11425903; doi:10.1002/advs.202403991)
Supplement: Supplementary file 1 — Supporting Information [file ADVS-11-2403991-s001.docx]

Supporting Information

Sterile diet causes gut microbiome collapse of cancer patients post hematopoietic cell transplantation, but normal diet recovers them

Wenqing Hong^1^†, Yun Wu^2^†, Zimin Sun^2,3^*, Shu Yang^4^*, Qing Cheng^2^, Huilan Liu^2^, Xiaoxing Lin^1^, Renjie Ni^1^, Yuping Yao^1^, Shuijing Wang^1^, Zihao Zheng^1^, Anyi Sun^1^, Chuanwu Xi^5^, Liyan Song^1,6^*

**Table S1.**

| Group1 | Group2 | P | *P* signif | Method |
| --- | --- | --- | --- | --- |
| F | S1 | 0.0621 | ns | Wilcoxon |
| F | S2 | 0.7412 | ns | Wilcoxon |
| F | S3 | 0.7925 | ns | Wilcoxon |
| F | S4 | 0.4672 | ns | Wilcoxon |
| F | S5 | 0.2436 | ns | Wilcoxon |
| F | S6 | 0.0084 | ** | Wilcoxon |
| F | S7 | 0.7117 | ns | Wilcoxon |
| F | S8 | 0.0315 | * | Wilcoxon |
| F | S9 | 0.385 | ns | Wilcoxon |
| S1 | S2 | 0.1551 | ns | Wilcoxon |
| S1 | S3 | 0.1869 | ns | Wilcoxon |
| S1 | S4 | 0.1097 | ns | Wilcoxon |
| S1 | S5 | 0.0114 | * | Wilcoxon |
| S1 | S6 | 0.712 | ns | Wilcoxon |
| S1 | S7 | 0.0244 | * | Wilcoxon |
| S1 | S8 | 0.7839 | ns | Wilcoxon |
| S1 | S9 | 0.1795 | ns | Wilcoxon |
| S2 | S3 | 0.9598 | ns | Wilcoxon |
| S2 | S4 | 0.3837 | ns | Wilcoxon |
| S2 | S5 | 0.2173 | ns | Wilcoxon |
| S2 | S6 | 0.0456 | * | Wilcoxon |
| S2 | S7 | 0.4553 | ns | Wilcoxon |
| S2 | S8 | 0.0924 | ns | Wilcoxon |
| S2 | S9 | 0.5195 | ns | Wilcoxon |
| S3 | S4 | 0.4799 | ns | Wilcoxon |
| S3 | S5 | 0.2122 | ns | Wilcoxon |
| S3 | S6 | 0.2325 | ns | Wilcoxon |
| S3 | S7 | 0.5056 | ns | Wilcoxon |
| S3 | S8 | 0.2319 | ns | Wilcoxon |
| S3 | S9 | 0.942 | ns | Wilcoxon |
| S4 | S5 | 0.7149 | ns | Wilcoxon |
| S4 | S6 | 0.1246 | ns | Wilcoxon |
| S4 | S7 | 0.5367 | ns | Wilcoxon |
| S4 | S8 | 0.1007 | ns | Wilcoxon |
| S4 | S9 | 0.3508 | ns | Wilcoxon |
| S5 | S6 | 0.0209 | * | Wilcoxon |
| S5 | S7 | 0.4891 | ns | Wilcoxon |
| S5 | S8 | 0.0188 | * | Wilcoxon |
| S5 | S9 | 0.1466 | ns | Wilcoxon |
| S6 | S7 | 0.0186 | * | Wilcoxon |
| S6 | S8 | 0.5824 | ns | Wilcoxon |
| S6 | S9 | 0.1226 | ns | Wilcoxon |
| S7 | S8 | 0.0188 | * | Wilcoxon |
| S7 | S9 | 0.193 | ns | Wilcoxon |
| S8 | S9 | 0.2649 | ns | Wilcoxon |

**Table S2.**

Missing sample information table. "*" indicates that the sample volume is insufficient to measure short-chain fatty acids (SCFAs), "**" indicates that the sample volume is insufficient to meet the sequencing requirements, the total number of samples sequenced in this study was 83, and the total number of samples measured for SCFAs was 58.

|  |  | Before  surgery |  | Sterile diet | | | |  | Normal diet | | | |  | Family  number |
| --- | --- | --- | --- | --- | --- | --- | --- | --- | --- | --- | --- | --- | --- | --- |
| Group |  | S1 |  | S2 | S3 | S4 | S5 |  | S6 | S7 | S8 | S9 |  | F |
| 1 |  | 1-1 |  | 1-2 | 1-3 | 1-4 | 1-5* |  | 1-6 | 1-7* | 1-8 | 1-9 |  | 1-10 |
| 2 |  | 2-1 |  | 2-2* | 2-3* | 2-4 | 2-5 |  | 2-6 | 2-7 | 2-8 | 2-9* |  | 2-10* |
| 3 |  | 3-1* |  | 3-2 | 3-3 | 3-4** | 3-5* |  | 3-6 | 3-7 | 3-8 | 3-9 |  | 3-10* |
| 4 |  | 4-1 |  | 4-2 | 4-3 | 4-4** | 4-5 |  | 4-6 | 4-7 | 4-8 | 4-9* |  | 4-10* |
| 5 |  | 5-1 |  | 5-2* | 5-3 | 5-4** | 5-5** |  | 5-6 | 5-7 | 5-8* | 5-9 |  | 5-10* |
| 6 |  | 6-1 |  | 6-2 | 6-3* | 6-4 | 6-5 |  | 6-6** | 6-7 | 6-8 | 6-9 |  | 6-10 |
| 7 |  | 7-1 |  | 7-2 | 7-3 | 7-4* | 7-5 |  | 7-6 | 7-7 | 7-8* | 7-9 |  | 7-10 |
| 8 |  | 8-1 |  | 8-2 | 8-3 | 8-4 | 8-5 |  | 8-6 | 8-7 | 8-8* | 8-9* |  | 8-10* |
| 9 |  | 9-1* |  | 9-2 | 9-3 | 9-4 | 9-5* |  | 9-6** | 9-7** | 9-8 | 9-9* |  | 9-10* |

**Table S3.**

The baseline data of patients

| **No.** | **Patient's gender /**  **age (years) / weight (kg)** | **Gender / age of relatives (years)** | **Diagnostic** | **Transplant disease status** | **Pre-treatment programmer** | **HLA donor-recipient compatibility** | **ABO（D/R）** | **TNC × 10^7^/kg** | **CD34^+^ cells × 10^5^ / kg** | **Plt ≥ 20 * 10^9^ / L (d)** | **ANC ≥ 0.5 * 10^9^ / L(d)** | **aGVHD onset time (d) / degree** | **Return / time (months)** | **Cause of death** |
| --- | --- | --- | --- | --- | --- | --- | --- | --- | --- | --- | --- | --- | --- | --- |
| 1 | F/3/19 | F/45 | AML | CR1 | Flu/Bu/CY | 4/6 | A/A | 3.58 | 4.69 | 19 | 14 | none | die / 5 | Cerebral hemorrhage |
| 2 | F/21/53 | M/45 | ALL | CR1 | Flu/Bu/CY | 4/6 | O/B | 2.59 | 1.68 | 41 | 16 | none | survival /69^+^ |  |
| 3 | F/25/74 | M/49 | AML | CR1 | Flu/Bu/CY | 4/6 | O/O | 3.47 | 2.08 | 34 | 15 | none | die /9 | Lung infection |
| 4 | F/7/19 | F/45 | ALL | CR3 | Flu/Bu/CY | 5/6 | O/AB | 6.96 | 2.92 | 32 | 22 | none | survival /69^+^ |  |
| 5 | F/13/40 | F/54 | ALL | CR2 | Flu/Bu/CY | 4/6 | B/O | 4.16 | 1.54 | 34 | 14 | 16 / Ⅰ degree | survival /69^+^ |  |
| 6 | M/28/56 | F/27 | ALL | CR1 | Flu/Bu/CY | 3/6 | A/B | 2.52 | 4.54 | 34 | 19 | none | survival /68^+^ |  |
| 7 | F/3/14 | F/24 | ALL | NR2 | Flu/Bu/CY | 5/6 | A/B | 9.05 | 3.44 | 20 | 18 | 27 / Ⅳ degree | survival /68^+^ |  |
| 8 | M/34/74 | F/33 | ALL | CR2 | Flu/Bu/CY | 5/6 | B/A | 3.44 | 1.34 | 39 | 19 | 25 / Ⅰ degree | die /30.2 | Recurrence of the original disease |
| 9 | M/6/20 | F/36 | AML | NR1 | Flu/Bu/CY | 5/6 | O/O | 6.67 | 2.2 | 41 | 19 | none | survival /68^+^ |  |

Note: ABO: ABO blood group system; ALL, acute lymphoblastic leukaemia; AML, acute myeloid leukaemia; Flu, fludarabine; Bu, busulfan; Cy, cyclophosphamide; M, male; F, female; CR1, first complete remission; CR2, second complete remission; NR, null remission; D, Donor; R, Recipient; TNC: total nucleated cells; Plt: platelet; ANC: Absolute Neutrophil Count; aGVHD: Acute graft-versus-host disease.

**Table S4.** The relative abundant of OTU 760 in every sample, sample points that do not appear indicate a relative abundance of OTU760 of 0.

| Sample ID | group | OTU760 |
| --- | --- | --- |
| 9_10 | F | 0.002% |
| 9_4 | S4 | 0.002% |
| 5_1 | S1 | 0.002% |
| 9_5 | S5 | 0.003% |
| 2_10 | F | 0.004% |
| 2_9 | S9 | 0.008% |
| 5_9 | S9 | 0.011% |
| 7_3 | S3 | 0.034% |
| 5_2 | S2 | 0.058% |
| 5_8 | S8 | 0.096% |
| 5_3 | S3 | 0.586% |
| 5_7 | S7 | 12.106% |
| 9_8 | S8 | 21.204% |
| 5_6 | S6 | 28.421% |


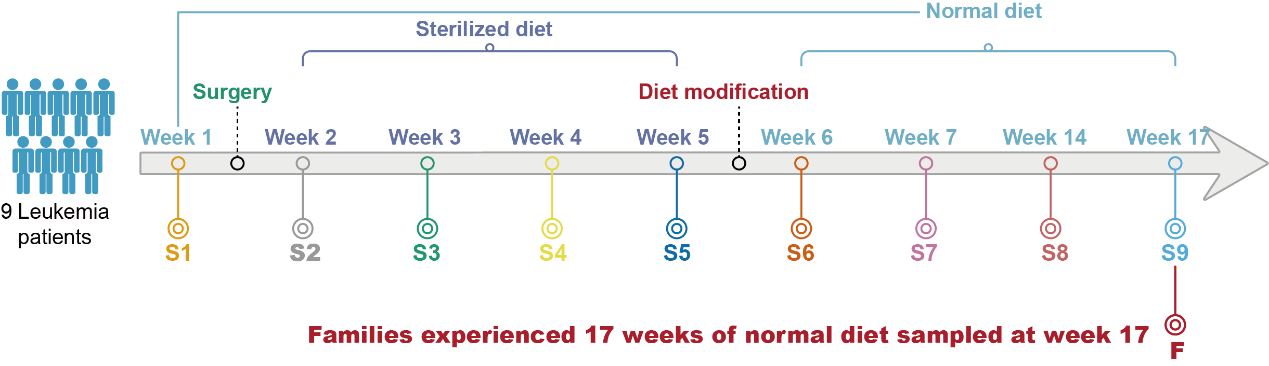


Figure S1.

Experimental design.


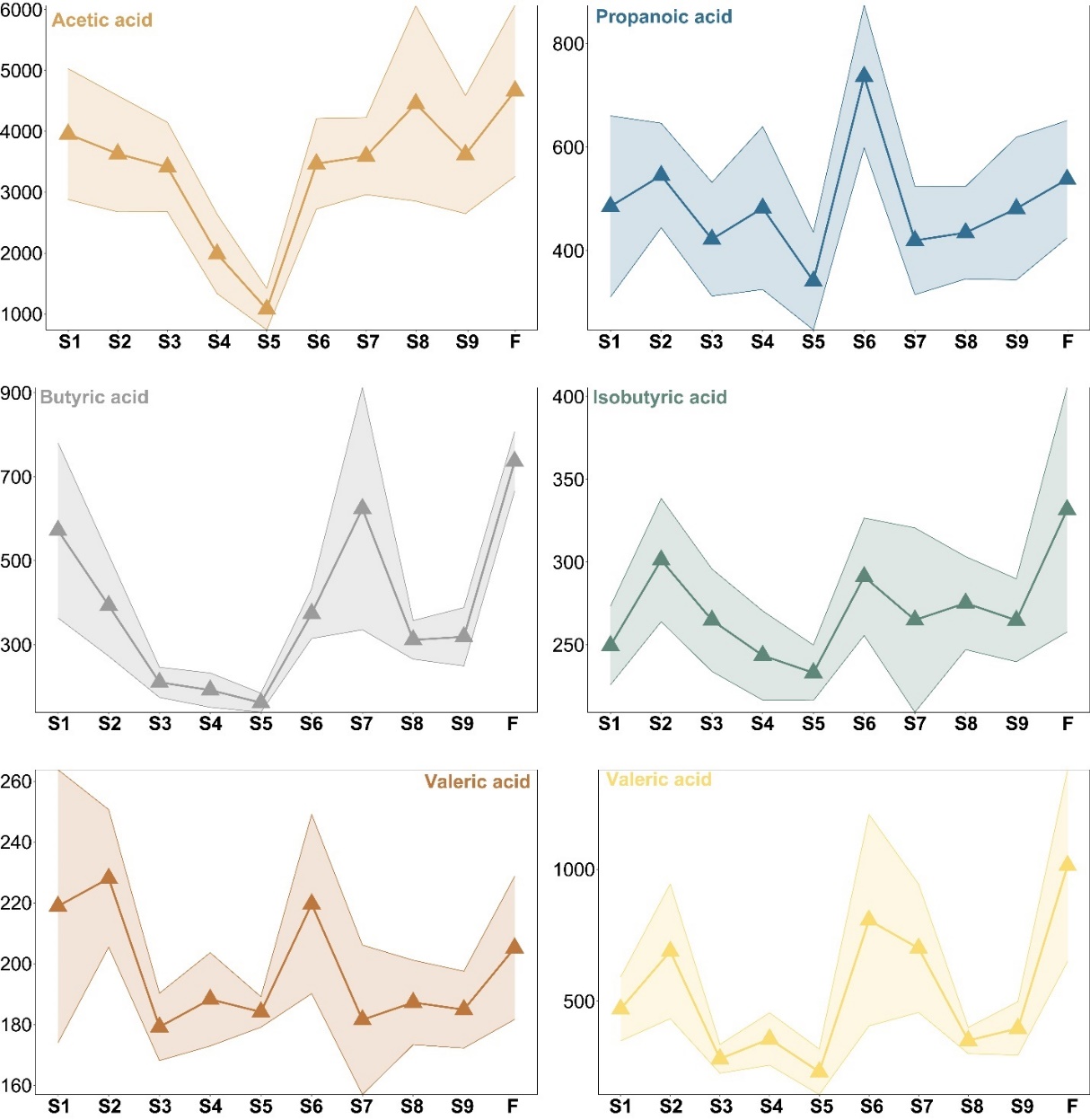


Figure S2.

Trends in short-chain fatty acids during dietary interventions, shaded areas indicate standard error of the mean.


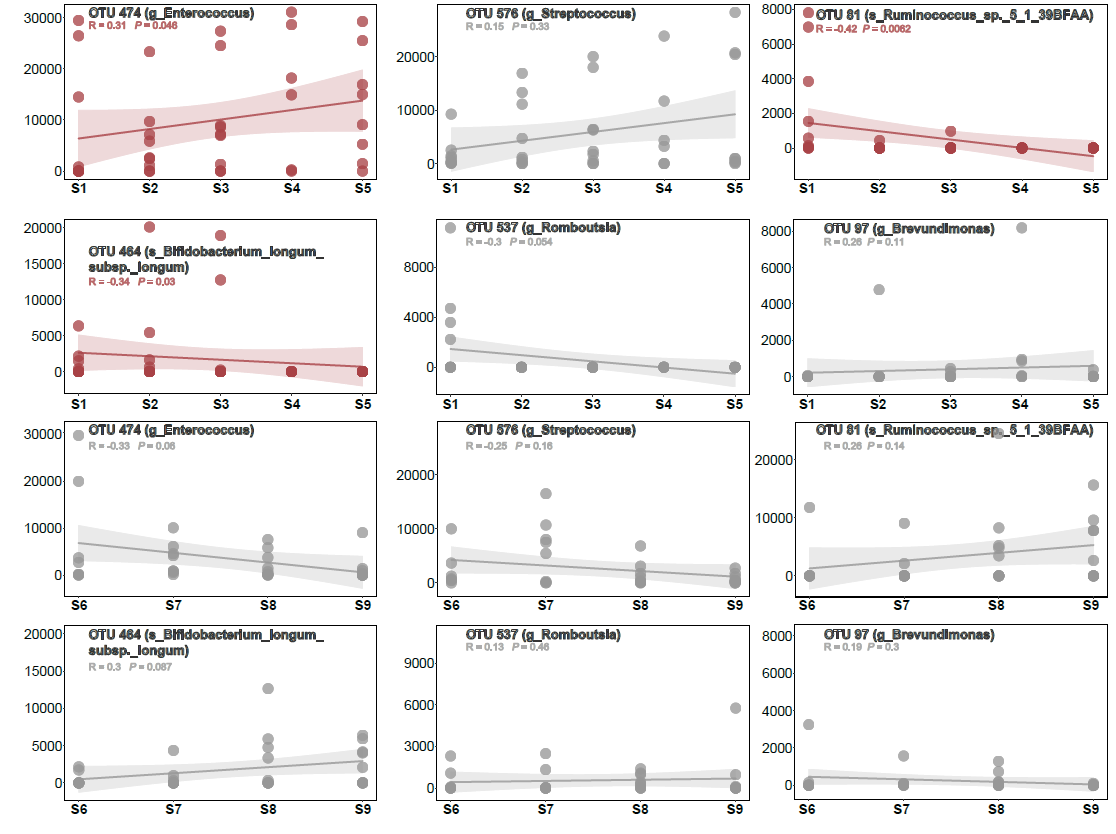


Figure S3.

Correlation of representative OTUs with sterile or normal diet, the shaded areas are 95% confidence interval, red color indicates correlation is statistics significant, Spearman test.


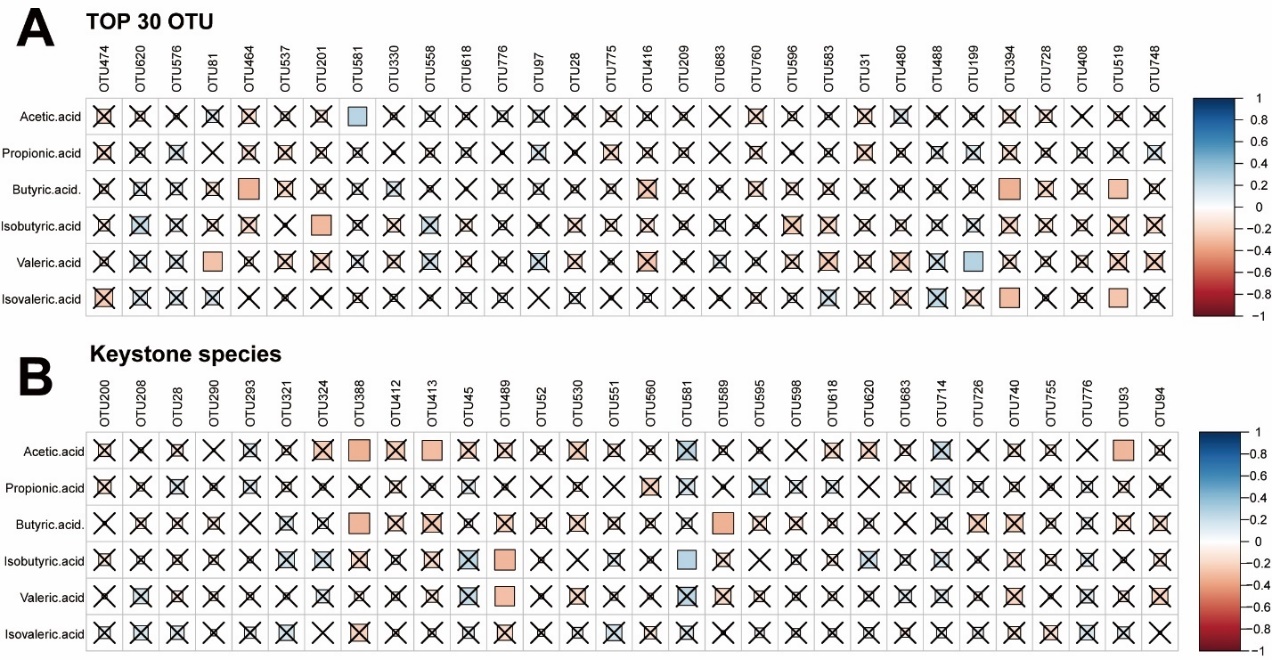


Figure S4.

Correlation of OTUs with short-chain fatty acid content for keystone species and Top 30 abundance, "×" indicates non-statistics significant (*P* > 0.05), Spearman test.


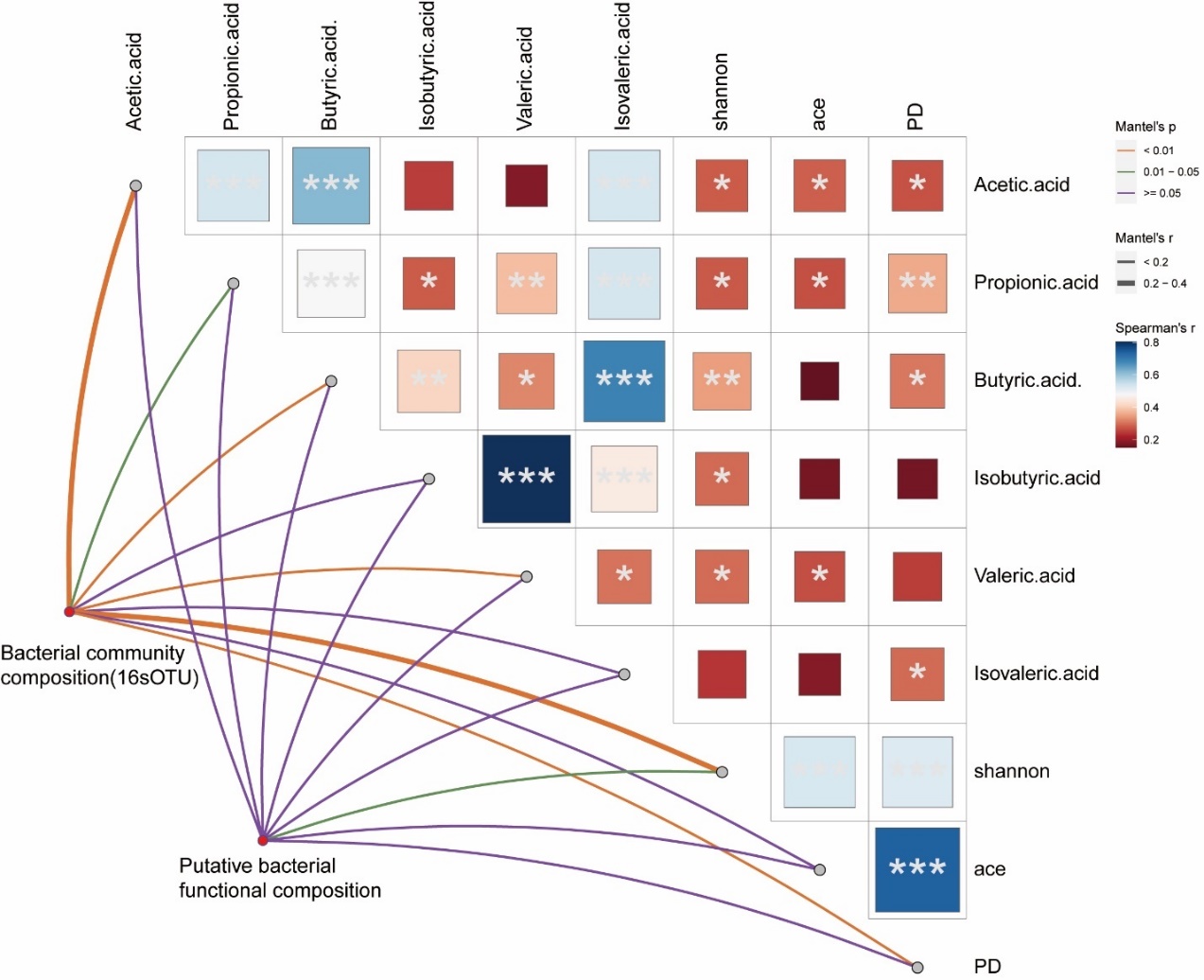


Figure S5.

Correlation test between gut microbial characteristics and fatty acids. Correlation between OTUs and KOs and fatty acids, significance was defined as **P* < 0.05, ***P* < 0.01, and ****P* < 0.001, the dashed line indicates that the R < 0, Spearman test.


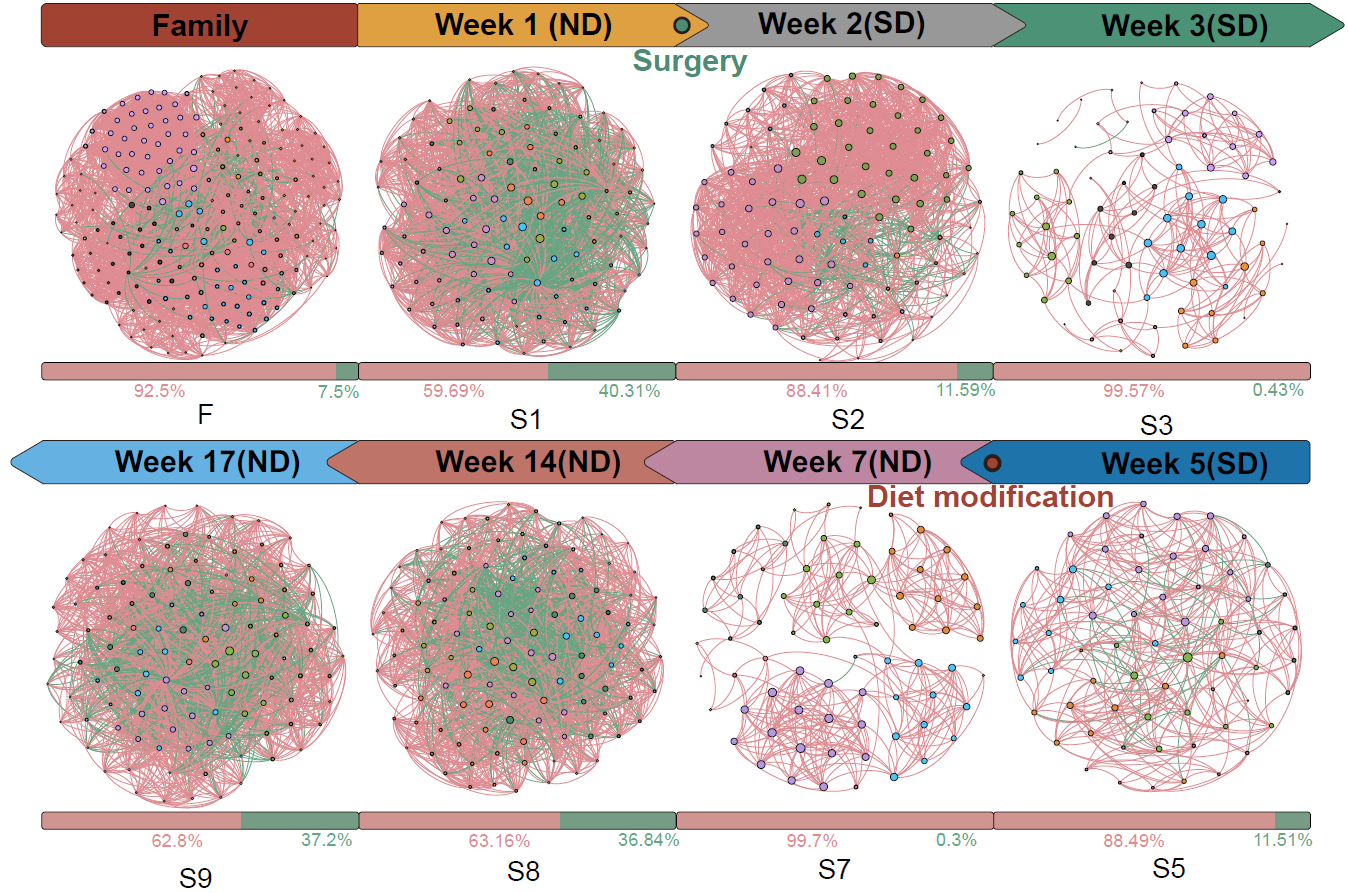


Figure S6.

Distribution and variation of positive and negative correlation effects between edges in the network, red edges indicate positive correlation, and green edges indicate negative correlation. **ND** denotes normal diet; **SD** denotes sterile diet.


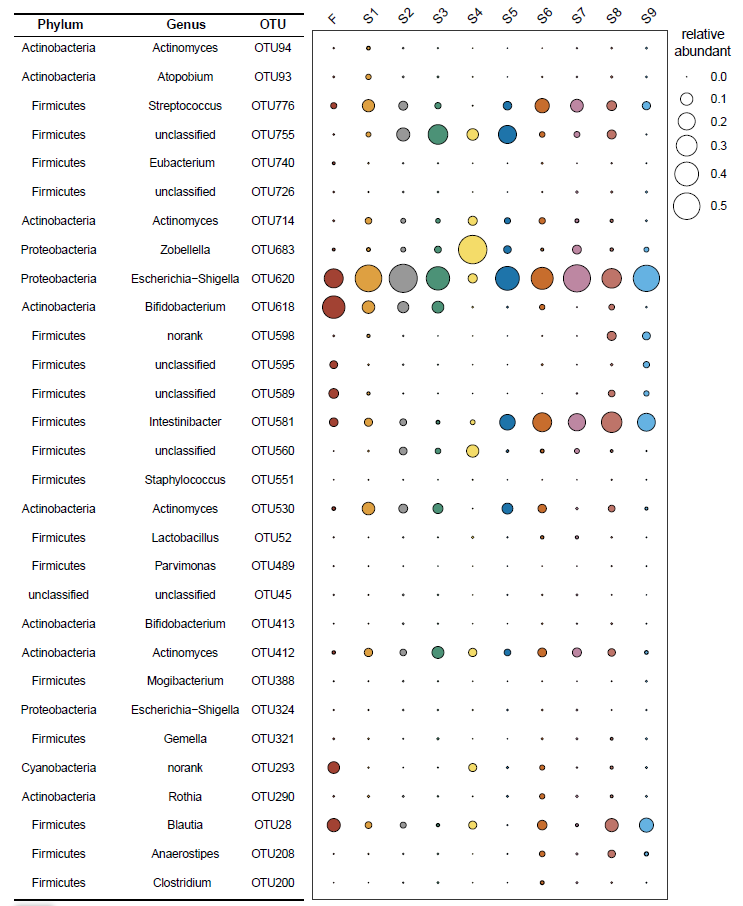


Figure S7.

Relative abundance of keystone species that were present in two or more networks.


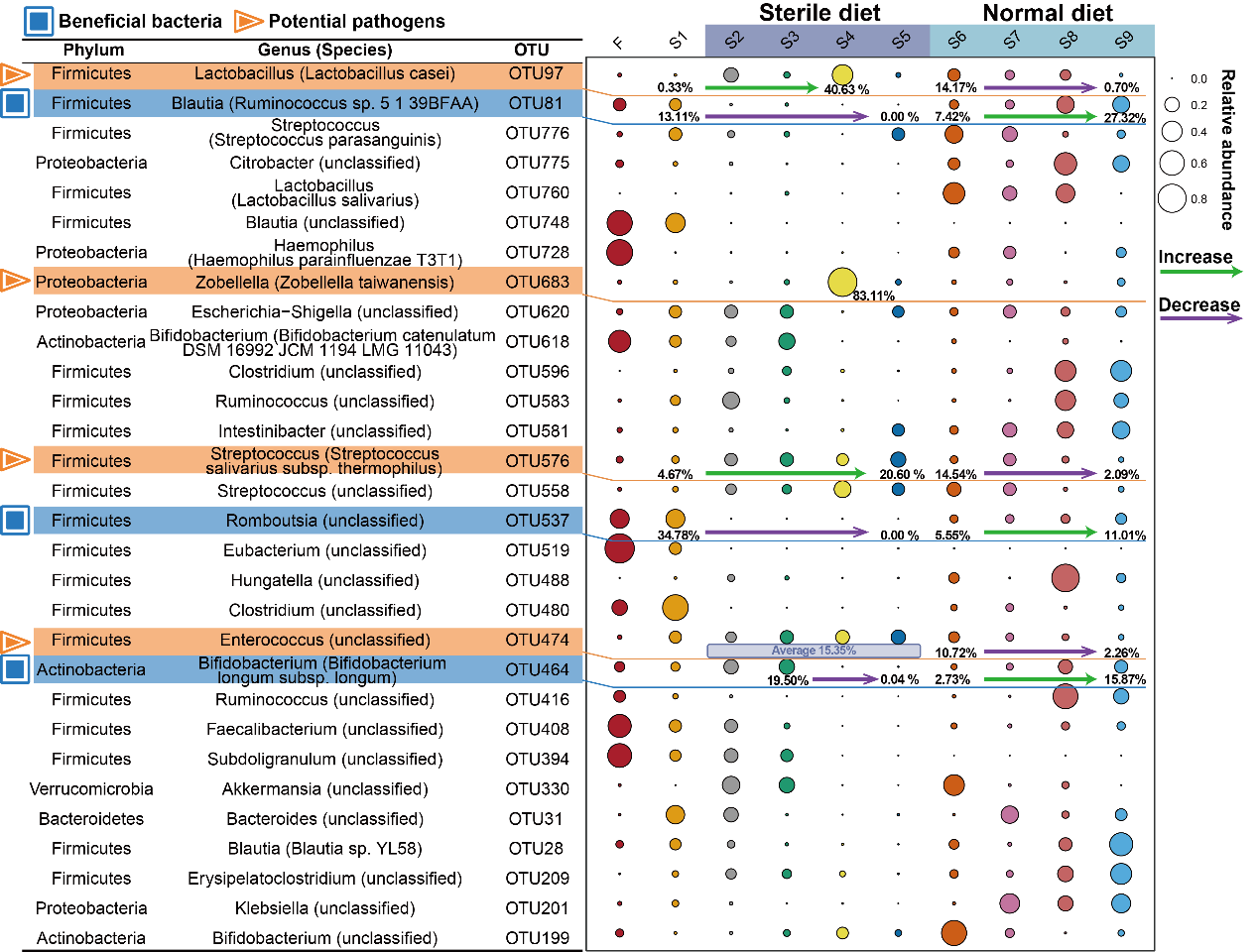


Figure S8.

Top 30 OTUs in terms of abundance, with species level information added.


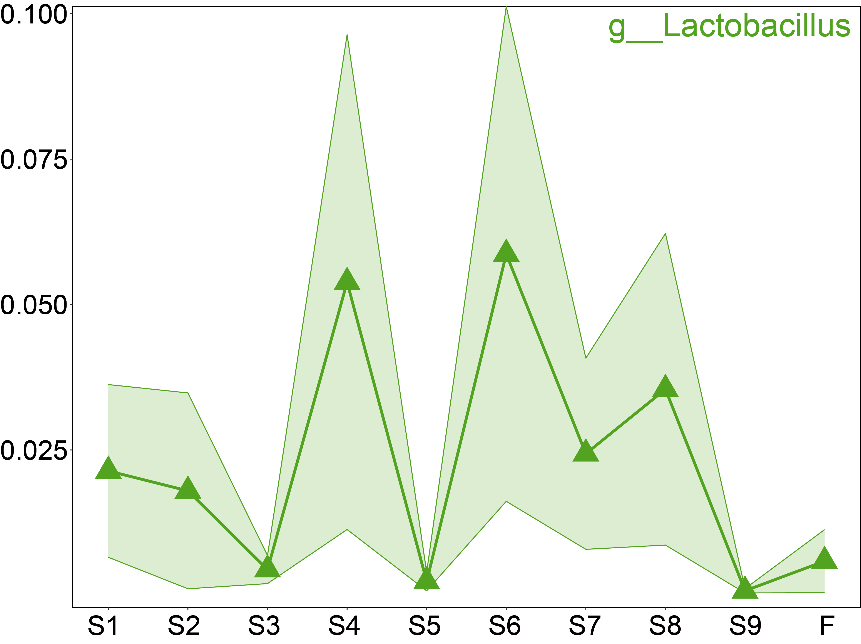


Figure S9.

Relative abundance distribution of the overall genus Lactobacillus.
